# Prognostic significance of Body Mass index in patients with localized renal cell carcinoma

**DOI:** 10.1590/S1677-5538.IBJU.2017.0629

**Published:** 2018

**Authors:** Chengtao Wang, Zebin Chen, Jun Dong, Bixiu Wen, Yong Fang

**Affiliations:** 1 Department of Radiation Oncology, First Affiliated Hospital of Sun Yat - Sen University, Guangzhou, China; 2 Department of Urology, First Affiliated Hospital of Sun Yat - Sen University, Guangzhou, China

**Keywords:** Body Mass Index, Carcinoma, Renal Cell, Prognosis

## Abstract

**Objective:**

To investigate the relationship between the pretreatment body mass index (BMI) and the clinical outcomes in patients with localized stage I - III renal cell carcinoma (RCC) surgically treated.

**Materials and Methods:**

From January 2000 to December 2012, 798 patients with stage I - III RCC were recruited from First Affiliated Hospital and Cancer Center of Sun Yat - Sen University. Patients were divided into two groups of BMI < 25 kg / m2 or BMI ≥ 25 kg / m2 according to the World Health Organization classifications for Asian populations. The differences in the long-term survival of these two BMI groups were analyzed.

**Results:**

The 5 - year failure - free survival rates for BMI < 25 kg / m2 and BMI ≥ 25 kg / m2 groups were 81.3% and 93.3%, respectively (P = 0.002), and the 5 - year overall survival rates were 82.5% and 93.8%, respectively (P = 0.003). BMI was a favored prognostic factor of overall survival and failure - free survival in a Cox regression model.

**Conclusions:**

Pretreatment body mass index was an independent prognostic factor for Chinese patients surgically treated, localized stage I - III RCC.

## INTRODUCTION

Renal cell carcinoma (RCC) is one of the most common urologic malignancies, and its incidence has steadily increased in recent decades. Several risk factors for developing of RCC have been reported, including smoking, hypertension and obesity (1).

Stratification of the patients with RCC into categories with different risk of local recurrence, progression and survival would improve the standard of preoperative patient's counseling and treatment planning. Several anatomical, clinical, histological and molecular variables can predict the probabilities of recurrence, progression, and both overall and cancer - specific survival of the patients with RCC (2).

The relationship between Body Mass Index (BMI) and the prognosis of cancer is not consistent. According to some published studies, a high BMI was associated with a favorable prognosis for various tumor types, including head and neck cancer (3), esophageal cancer (4), colon cancer (5) and endometrial cancer (6). However, the results of some studies showed that patients with a higher BMI had a worse prognosis for breast cancer (7), and prostate cancer (8). In renal cell carcinoma, obesity seems to increase the risk of developing RCC (1).

Whereas, more recent studies conducted in Western countries indicate that obese patients treated with surgery for RCC may have a more favorable prognosis than patients with normal BMI (9-12). It is unknown whether the same associations are seen in Chinese patients, who have a different body composition from White and Black populations. As we all known, different ethnic groups may show clinically significant increases in fat composition or glucose at lower BMI than those predicted in established BMI cut points.

Therefore, the aim of this study was to assess the influence of BMI, using the World Health Organization (WHO) categories recommended for Asians (13), on treatment results in Chinese patients with surgically treated, localized stage I - III RCC.

## MATERIALS AND METHODS

### Patients

By using the departmental surgical database of our two institutions (First Affiliated Hospital and Cancer Center of Sun Yat - Sen University), we identified 798 patients aged 18 years old who were treated with radical nephrectomy for unilateral, sporadic localized stage I - III RCC between 2000 and 2012. Data collected from each patient included age at diagnosis, gender, pretreatment BMI, pretreatment hemoglobin (Hb), pretreatment alkaline phosphatase (ALP), pretreatment platelets (PLT), TNM stage, histological subtype and survival time. BMI was calculated as the patient's weight on day 1 of admission (in kilograms) divided by the patient's height squared (in meters).

Tumors were classified in accordance with the 2002 TNM staging system. Histological subtypes were stratified in accordance with the 2002 AJCC / UICC classification, and only tumors of clear - cell, chromophobe, and papillary histology were included. The BMI was categorized based on WHO recommendations for Asians.

### Statistical analysis

All events were measured from the date of surgery, and statistical tests were performed using SPSS V17.0 (SPSS Inc., Chicago, IL). The actuarial rates were calculated with the Kaplan - Meier method, and the differences were compared with the log - rank test. The time to the first defining event was assessed for the following endpoints: failure free survival (FFS - disease failure at any site), and overall survival (OS - all cause mortality). The survival rates were calculated using the Kaplan - Meier method and compared with the log - rank test. A 2 - tailed P value of less than 05 was considered statistically significant.

The entire cohort was analyzed using the Cox proportional hazards model for OS and FFS. Potentially important prognostic factors considered in the modeling process were patient gender (male vs. female), age (≥ 50 years vs < 50 years), symptoms at presentation (yes vs. no), histology (chromophobe vs. papillary vs clear cell), pTNM stage (III vs. II vs. I), Hb (non - anemia vs. anemia), PLT (> 300 vs. ≤ 300), ALP (> 70 vs. ≤ 70), tumor necrosis (yes vs. no) and BMI (≥ 25 kg / m^2^ vs. < 25 kg / m^2^).

The last follow-up visit was in June 2015, with a median follow-up period of 46 months.

## RESULTS

Table-1 summarizes the clinical and pathologic characteristics of 798 patients according to the WHO BMI subgroups. The mean age was 51 years (range: 19 – 84 years) and the mean BMI was 23.8 kg / m^2^ (range: 14.4 – 41.7 kg / m^2^) for the entire group. Three hundred and thirty - seven (42.2%) patients had a BMI less than 25 kg / m^2^, and 461 (51.2%) had a BMI equal or greater than 25 kg / m^2^. When comparing risk parameters between BMI categories, the two BMI groups showed similar demographics, such as in the age, histology, pTNM stage, ALP and tumor necrosis. Aside from these factors, gender, symptoms at presentation, pretreatment Hb and PLT were significantly different. There were more female patients and patients with symptoms at presentation in BMI less than 25 group. Patients with a BMI less than 25 were significantly more likely to have lower pretreatment Hb and higher pretreatment PLT (P = 0.003, P = 0.017, respectively; Table-1). Laparoscopic partial nephrectomy was performed in 39 (4.9%) patients, radical nephroureterectomy was performed in 435 (54.5%) patients, open partial nephrectomy completed in 183 patients (22.9%), and 141 of them had a laparoscopic radical nephrectomy (17.7%). The distribution of surgery modality was balanced in both BMI groups.

**Table 1:** Baseline Characteristics by BMI Group.

Characteristics			Total (%)	BMI Group		P value
				<25 (%)	≥ 25 (%)	
Case (Percentage)			798(100)	337(42.2)	461(57.8)	
Mean BMI			23.8	20.7	26.0	
[mean(range)]			(14.4-41.7)	(14.4-22.9)	(23-41.7)	
Age(y) [mean(range)]			51 (19-84)	51(19-81)	52 (19-84)	
		<50 year	383 (48.0)	168(49.9)	215(46.6)	0.369
		≥ 50 year	415 (52.0)	169(50.1)	246(53.4)	
**Sex**						<0.001
		Male	545 (68.3)	206 (61.1)	339 (73.5)	
		Female	253 (31.7)	131 (38.9)	122 (26.5)	
**Symptoms at presentation**						0.008
		Yes	296 (37.1)	143 (42.4)	153 (33.2)	
		No	502 (62.9)	194 (57.6)	308 (66.8)	
**Histology**						0.406
	Clear cell renal carcinoma		720 (90.2)	302(89.6)	418(90.7)	
	Papillary renal cell carcinoma		43 (5.4)	22(6.5)	21(4.6)	
	Chromophobe renal carcinoma		35 (4.4)	13(3.9)	22(4.8)	
	pTNM stage					0.070
		I	596 (74.7)	238(70.6)	358(77.7)	
		II	171 (21.4)	85(25.2)	86(18.7)	
		III	31 (3.9)	14(4.2)	17(3.7)	
**Hb**						0.003
	Male: Hb<120; Female: Hb<110		106 (13.3)	59(17.5)	47(10.2)	
	Male: Hb ≥ 120; Female: Hb ≥ 110		692 (86.7)	278(82.5)	414(89.8)	
**PLT**						0.017
		≤ 300	690 (85.5)	280(83.1)	410(88.9)	
		>300	108 (13.5)	57(16.9)	51(11.1)	
**ALP**						0.894
		≤ 70	445 (55.8)	187(55.5)	258 (56.0)	
		>70	353 (44.2)	150(44.5)	203 (44.0)	
**Tumor necrosis**						0.660
		Yes	111 (13.9)	288 (85.5)	399 (86.6)	
		No	687 (86.1)	49 (14.5)	62 (13.4)	

**BMI** = body mass index; Hb = hemoglobin; **PLT** = platelets; **ALP** = alkaline phosphatase

**Figure 1:**
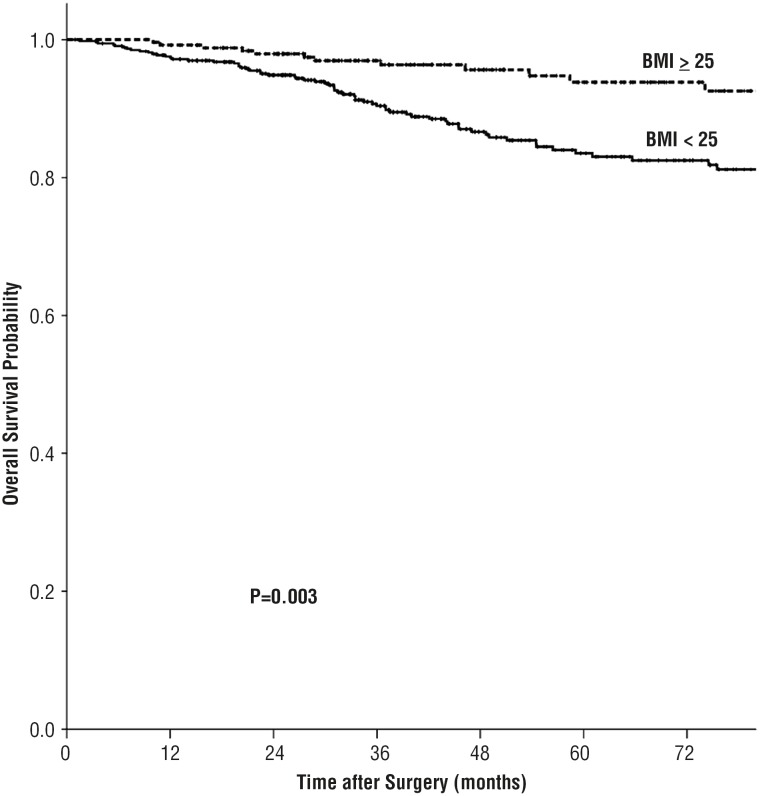
Overall survival by BMI group among Chinese patients with RCC.

**Figure 2:**
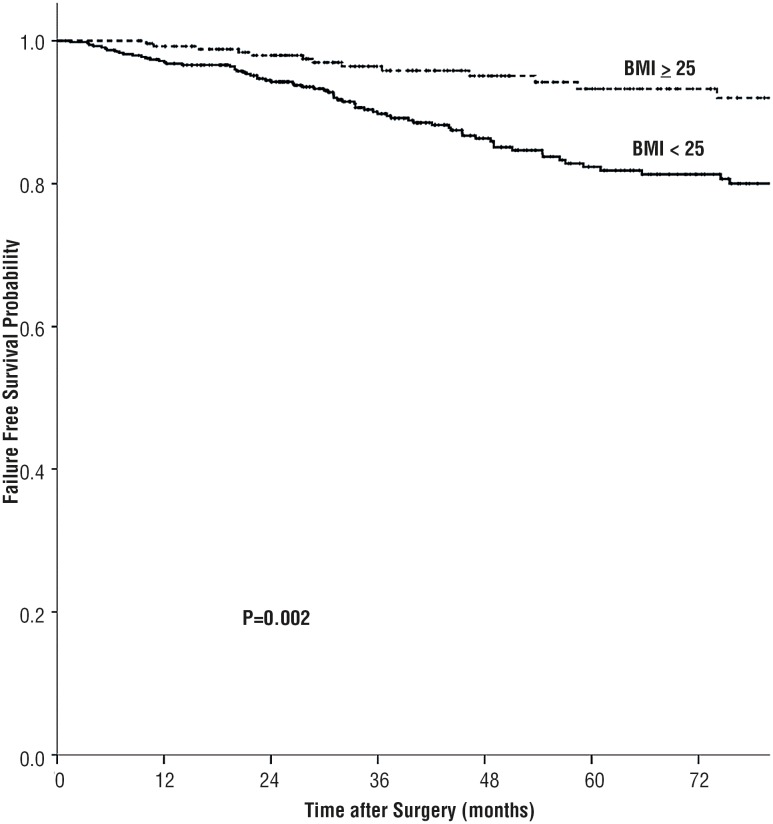
Failure Free Survival by BMI group among Chinese patients with RCC.

Surgery complications were as follow: hemorrhage and hematoma occurred in 21 patients; peritoneal injury in 36 patients; abdominal organ injury in 5 patients; vascular injury in 13 patients; urinary fistula in 7 patients; pleura injury in 3 patients; wound infection in 8 patients and severe hypercapnia in 2 patients. At a median follow-up period of 46.0 months, 11.2% (89 / 798) patients developed tumor progression (31 with tumor recurrence, 58 with distant metastasis) and10.5% (84 / 798) patient were dead. The 5 - year failure - free survival rates for BMI < 25 kg / m^2^ and BMI ≥ 25 kg / m^2^ groups were 81.3% and 93.3%, respectively (P = 0.002) and the 5 - year overall survival rates were 82.5% and 93.8%, respectively (P = 0.003). Lower BMI was associated significantly with poor prognosis. Patients with preoperative BMI less than 25 kg / m^2^ had a significantly reduced rate of survival than those BMI equal or greater than 25 kg / m^2^ with regard to FFS and OS (Figures 1 and 2).

Univariable analyses of the factors influencing FFS and OS are shown in Table-2. Univariable analysis demonstrated that the presence of symptoms at presentation, age, pTNM stage, tumor necrosis, non - anemia, ALP, BMI and thrombocytosis were significant predictors of FFS and OS (Table-2).

**Table 2:** Pretreatment BMI Effect on Different Endpoint: univariate Analysis in Cox Regression Model.

Characteristics	FFS			OS		
	HR	95% CI	p value	HR	95% CI	p value
Gender (male vs female)	1.50	0.92-2.45	0.102	1.38	0.84-2.26	0.201
Age (≥ 50 years vs<50 years)	1.99	1.27-3.10	0.002	2.03	1.28-3.21	0.003
Symptoms at presentation (yes vs no)	2.81	1.83-4.32	0.000	2.94	1.88-4.58	0.000
Histology(Chromophobe vs Papillary vs Clear cell)	1.56	0.75-3.25	0.233	1.63	0.77-3.45	0.207
pTNM stage(III vsIIvsI)	3.36	2.53-4.47	0.000	3.12	2.34-4.18	0.000
Hb (non-anemia vs anemia)	0.30	0.19-0.48	0.000	0.27	0.17-0.42	0.000
PLT (>300 vs ≤ 300)	2.68	1.69-4.25	0.000	2.89	1.81-4.61	0.000
ALP (>70 vs ≤ 70)	5.89	2.84-12.2	0.000	3.65	2.22-5.99	0.000
Tumor necrosis (yes vs no)	3.08	1.98-4.78	0.000	3.22	2.05-5.05	0.000
BMI Group (≥ 25 kg/m^2^ vs <25 kg/m^2^)	0.45	0.27-0.77	0.003	0.45	0.26-0.78	0.004

**BMI** = body mass index; **Hb** = hemoglobin; **PLT** = platelets; **ALP** = alkaline phosphatase

Stepwise multivariable analysis showed that BMI (HR, 0.54; P = 0.029) was an independent predictor of OS, along with the presence of symptoms at presentation (HR, 1.68; P = 0.031), pTNM stage (HR, 2.30; P < 001), age (HR, 1.72; P = 0.023) and non-anemia (HR, 0.55; P = 0.025) (Table-3). Stepwise multivariable analysis showed that BMI (HR, 0.53; P = 0.022) was an independent predictor of FFS, along with the presence of symptoms at presentation (HR, 1.60; P = 0.043), pTNM stage (HR, 2.59; P < 001), age (HR, 1.75; P = 0.016) and non - anemia (HR, 0.60; P = 0.045) (Table-3).

**Table 3:** Pretreatment BMI Effect on Different Endpoint: Multivariate Analysis in Cox Regression Model.

Characteristics	FFS			OS		
	HR	95% CI	p value	HR	95% CI	p value
Age (≥50 years vs<50 years)	1.75	1.11-2.75	0.016	1.72	1.08-2.76	0.023
Symptoms at presentation (yes vs no)	1.60	1.02-2.52	0.043	1.68	1.05-2.69	0.031
pTNM stage(III vs II vs I)	2.59	1.83-3.64	0.000	2.30	1.62-3.28	0.000
Hb (non-anemia vs anemia)	0.60	0.36-0.99	0.045	0.55	0.33-0.93	0.025
PLT (>300 vs ≤ 300)	1.22	0.72-2.08	0.456	1.33	0.77-2.27	0.306
ALP (>70 vs ≤ 70)	2.04	0.91-4.57	0.083	1.94	0.86-4.37	0.111
Tumor necrosis (yes vs no)	1.29	0.78-2.11	0.321	1.38	0.83-2.30	0.210
BMI Group (≥ 25 kg/m^2^ vs < 25 kg/m^2^)	0.53	0.31-0.91	0.022	0.54	0.31-0.94	0.029

**BMI** = body mass index; **Hb** = hemoglobin; **PLT** = platelets; **ALP** = alkaline phosphatase

## DISCUSSION

To our knowledge, several epidemiological studies have suggested that obesity is a risk factor for the development of RCC (1, 11, 14). Due to the high rate of comorbidities, obesity is frequently considered to represent a major risk factor for complications after surgery (15). Previous reports have identified postoperative complications correlating with a high BMI (16-18). However, there are conflicting data relating obesity as a risk factor affecting overall or progression - free survival. Since 1991 when Yu et al. found a paradoxical positive association between obesity and overall and disease - free survival, there have been no prospective studies further evaluating this finding (19). A more contemporary retrospective review of 400 patients undergoing nephrectomy for RCC by Kamat et al. appears to confirm a more favorable prognosis and disease specific survival in overweight and obese patients when compared to normal weight patients (20). With regard to urologic neoplasms, it was shown that a high BMI does not affect oncologic outcomes after surgery. All these studies were conducted in Western countries. The body composition profile of Asian populations differs from that of white and black populations (13). Some studies conducted in Japan and Korea also demonstrated this phenomenon (12, 21). The present study is the first to investigate the influence of obesity on RCC prognosis in a Chinese population. In this study, we examined the association between BMI and other clinical / pathological characteristics, and evaluated the prognostic association of BMI with FFS and OS in Chinese patients with RCC who underwent radical or partial nephrectomy. We found pretreatment BMI was a favorable prognostic factor for Chinese patients with stage I - III RCC. Although obesity predisposed to an increased risk for developing RCC, the prognosis for obese patients treated with surgery was no worse and possibly better than normal weight subjects.

In this retrospective study, clinical / pathological factors significantly impacting FFS and OS for the study population were similar to previously published factors including age older than 50 years at surgery, symptoms at presentation, pTNM stage. Obese patients were more likely to have favorable clinical and pathologic conditions at diagnosis, including younger, less symptoms at presentation, lower stage, lower PLT and lower anemia when compared with under - to - normal weight patients. We therefore carefully adjusted for age, pTNM stage, symptom presence, baseline Hb, ALP and PLT, which may be related to patient survival. Although adjustment for other important risk factors associated with survival weakened the association for both OS and FFS, the association between obesity and RCC prognosis remained strong and highly significant. Being obese at the time of surgery might have a positive prognostic effect in patients. This result is in accordance with the retrospective studies. Parker et al. evaluated 970 patients with RCC and were unable to identify obesity (BMI ≥ 30 kg / m^2^) as a prognostic factor (HR 0.90, 0.65 – 1.23, P = 0.488) for CSS in their multivariate analysis, which also included the prognostic factors: Mayo Clinic Stage, size, TNM stage groups, nuclear grade and tumor necrosis. They concluded that BMI offers little additional prognostic information beyond the accepted prognostic features (10, 22). Being obese at the time of surgery might have a positive prognostic effect in patients.

The mechanism by which preoperative obesity may improve RCC survival is not well understood, although mechanisms linking obesity with RCC incidence have long been studied (23). RCC is a heterogeneous and complex disease (24), and the histologic subtypes of RCC differ with respect to genetic, pathologic, and clinical parameters (25-27). On the basis of this evidence, the relationship between obesity and RCC prognosis might be subtype specific. Furthermore, recent studies have shown that the association between obesity and the risk of developing RCC is subtype specific (28-30). Nevertheless, in the previous studies assessing the association between obesity and RCC survival, histologic subtype has been considered as a simple variable that is divided into two groups, cRCC and non - cRCC (31-34), or only patients with cRCC were included (9, 35). The relationship between obesity and RCC prognosis might be subtype specific in our study was not significant.

Some protein factors and signals in adipose tissue that suppress RCC progression have been reported (36). For example, adipose tissue synthesize leptin and the circulating levels of leptin are strongly related to obesity. Leptin has also been shown to play an important role in stimulating pro - inflammatory T helper 1 immune responses (37). In contrast, a change in the predominant immunologic response from T helper 1 to T helper 2 has been reported to correlate with increasing RCC stage (38). Therefore, as proposed by Rasmuson et al., leptin might play a pivotal role in delaying RCC progression (39). Another study showed an association between preoperative nutritional deficiency and poor OS and disease - free survival in RCC patients who underwent renal surgery (40). Patients with higher BMI, who generally have large appetites and high lipid concentrations, may adequately preserve their fat and muscle mass, thus allowing better nutritional status and potential survival advantage (41, 42). It may be plausible that obesity indicates favorable general health condition rather than it being responsible for improved outcomes.

The present study had some weaknesses. First, the study was retrospective, and the median follow-up time of 46.0 months for patients still alive was short. Second, our study lacked a central review of pathology, although most of the large multicenter studies did. Instead, urologic pathologists reviewed all specimens at each institution. Third, we could not assess potential prognostic factors, such as smoking, molecular markers, and sarcomatoid features in all patients. These factors may allow the identification of patients at high risk and affect the prognosis. However, our study includes the most widely accepted independent prognostic factors of nonmetastatic RCC, including age, pTNM stage, and tumor necrosis. Last, all of our study subjects were Chinese, and the distribution of BMI or cut - off value for Asian populations may be different than those for Western populations. Therefore, our results may not be directly applicable to Western populations. Taken together, a multi - institutional prospective study with a large number of patients would be required to confirm the present finding. Furthermore, basic biologic research would be needed to explain the contradictory effects of BMI on the risk and prognosis of RCC.

In conclusion, these findings suggest that pretreatment high BMI prior to renal surgery is associated with improved OS, FFS when compared with low BMI in Chinese population. This evidence may provide new insight into the effects of preoperative high BMI on improvements in RCC survival, and this could help physicians in predicting overall prognosis. Further research is needed to explain the biological mechanisms responsible for the benefit of high BMI on improved RCC survival, and to determine whether other modifiable lifestyle factors contribute to RCC survival.
